# Valorisation of spent activated carbon for accelerated compost stabilisation and enrichment of plant growth-promoting bacterial taxa

**DOI:** 10.1007/s11274-026-05223-2

**Published:** 2026-08-27

**Authors:** Halimatul Aliyah Othman, Mohd Hafif Samsudin, Mohammed Abdillah Ahmad Farid, Ahmad Muhaimin Roslan, Toshinari Maeda, Mohd Huzairi Mohd Zainudin, Hendrix Yulis Setyawan, Mohd Zulkhairi Mohd Yusoff

**Affiliations:** 1 Department of Bioprocess Technology, Faculty of Biotechnology and Biomolecular Sciences, Universiti Putra Malaysia, UPM Serdang, Selangor 43400 Malaysia; 2 Laboratory of Biopolymer and Derivatives, Institute of Tropical Forestry and Forest Products (INTROP), Universiti Putra Malaysia, UPM Serdang, Selangor 43400 Malaysia; 3 Department of Biological Functions Engineering, Graduate School of Life Science and Systems Engineering, Kyushu Institute of Technology, 2-4 Hibikino, Wakamatsu, Kitakyushu, Fukuoka 808-0196 Japan; 4 Laboratory of Sustainable Animal Production and Biodiversity, Institute of Tropical Agriculture and Food Security (ITAFoS), Universiti Putra Malaysia, UPM Serdang, Selangor 43400 Malaysia; 5 Department of Agroindustry, Faculty of Agricultural Technology, University of Brawijaya, Veteran St, Malang, East Java 65145 Indonesia

**Keywords:** Waste valorisation, Spent activated carbon, Co-composting, Chicken manure, Mushroom substrate, Plant growth-promoting bacteria

## Abstract

The sustainable management of spent activated carbon (SAC) from water treatment remains an environmental challenge because reuse options are limited and disposal still relies on landfilling. This study evaluated SAC as a carbonaceous amendment during co-composting of chicken manure and spent mushroom substrate at 0% (Control), 10% (T1), 20% (T2), and 30% (T3) addition levels. SAC addition shortened the thermophilic phase from 12 days in the Control to 9 days in T2 and T3, and SAC-amended treatments reached operational stabilisation within 30–42 days, as indicated by temperature decline, lower late-stage gas emissions, and Solvita CO₂/NH₃ indices. Increasing SAC dosage raised total organic carbon from 37.94% to 54.12% and compost yield from 41.94% to 54.70%, whereas total nitrogen concentration decreased from 2.28% to 1.34% because of dilution by recalcitrant carbon. Lower NH₃ emissions in the SAC-amended treatments were consistent with reduced volatilisation, although nitrogen retention was not quantified by a mass balance. All SAC-amended treatments achieved germination indices above 100% (111.48–129.87%), indicating the absence of phytotoxicity in the assay. High-throughput 16 S rRNA gene sequencing showed treatment- and stage-dependent shifts in bacterial community structure. At Day 60, T1 recorded the highest observed richness (197 OTUs) and Faith PD (23.14), whereas T2 had the highest combined relative abundance (22.47%) of bacterial taxa previously associated with plant growth-promoting functions, including members of Rhizobiaceae and the genera *Mesorhizobium* and *Ensifer*. Because Shannon diversity in the SAC-amended treatments did not exceed that of the Control at maturity, the results indicate selective community restructuring and enrichment rather than a uniform increase in bacterial diversity. Overall, moderate SAC addition accelerated compost stabilisation and promoted favourable bacterial succession, supporting a circular pathway for converting multiple waste streams into biologically enriched compost.

**Supplementary Information:**

The online version contains supplementary material available at https://doi.org/10.1007/s11274-026-05223-2.

## Introduction

The poultry sector continues to expand in response to rising protein demand and changing dietary patterns. In Malaysia, poultry stocks exceeded 284.6 million birds in 2021, illustrating the scale of associated waste generation (DVS, 2021). A single chicken produces approximately 80–100 g of manure per day; at commercial production scales, this creates a substantial waste-management challenge (Manogaran et al. 2022). If unmanaged, direct disposal or unregulated land application of chicken manure can cause ammonia volatilisation, odour, pathogen dissemination, nutrient imbalance, and greenhouse-gas emissions, thereby threatening environmental quality and public health (Hafiz Jamaludin et al. 2023; Manogaran et al. 2022).

Composting is an effective and scalable process that converts unstable organic waste into a safer, humus-like material through aerobic microbial decomposition across mesophilic, thermophilic, cooling, and maturation stages (Lin et al. 2022; Manogaran et al. 2022). The process reduces pathogen loads and phytotoxicity while producing an amendment that can improve soil fertility. Conventional chicken-manure composting nevertheless faces prolonged maturation, ammonia volatilisation, greenhouse-gas emissions, inadequate aeration, and variable product quality because chicken manure has a low carbon-to-nitrogen ratio and high moisture content (Manea et al. 2024). Co-composting with lignocellulosic bulking agents can improve porosity and moisture distribution. Spent mushroom substrate (SMS) is widely available and has been reported to improve composting efficiency, product quality, rice seedling growth, and suppression of soil-borne pathogens (Li et al. 2024; Zeng et al. 2023). Further optimisation is required to shorten the active phase, limit nitrogen loss, and improve the biological quality of the final compost.

Carbon-rich amendments, including biochar and activated carbon, have attracted interest because they can modify aeration and moisture conditions, retain ammonium, and provide microbial colonisation sites (Parra-Orobio et al. 2023; Sanchez-Monedero et al. 2018; Stacey et al. 2024). The addition of 20% biochar has also been reported to shift nitrifying and denitrifying bacterial communities and nitrogen transformation during composting (Zainudin et al. 2020). Carbonaceous amendments can therefore alter temperature profiles, nitrogen dynamics, and agronomic performance (Oviedo-Ocaña et al. 2025; Parra-Orobio et al. 2023; Stacey et al. 2024). Most previous work, however, has examined virgin carbon materials rather than post-use adsorbents.

Unlike virgin activated carbon, SAC has already undergone adsorption, which can partially occupy pores and modify surface functional groups through retained organic and inorganic constituents. These changes may affect nutrient adsorption, gas exchange, microbial attachment, and the availability of colonisation sites during composting. Consequently, the behaviour of SAC cannot be inferred directly from studies of virgin carbonaceous materials (Fouda-Mbanga et al. 2024; Renu and Sithole 2024). Although SAC reuse offers a circular waste-management opportunity and activated carbon can alter bacterial community structure in soil (Meynet et al. 2012), evidence specific to manure-based composting is limited, particularly for compost stabilisation, maturity, phytotoxicity, bacterial succession, and enrichment of taxa associated with plant growth-promoting functions.

This study evaluates the effects of SAC dosage on chicken manure–SMS co-composting by integrating temperature, moisture, pH, oxygen, carbon dioxide, maturity indices, compost quality, phytotoxicity, and high-throughput 16 S rRNA gene sequencing. By assessing post-use SAC rather than virgin biochar or activated carbon, the study tests whether SAC addition is associated with faster compost stabilisation and favourable bacterial succession while identifying dosage-dependent trade-offs in nutrient content and agronomic quality.

## Materials and methods

### Raw material preparation

Chicken manure (CM) was collected from a poultry farm at Universiti Putra Malaysia, SMS from Taman Pertanian Universiti, and SAC from a local water-treatment facility. Stones, feathers, and other foreign materials were removed manually. The SMS was homogenised and air-dried under ambient conditions for 24 h to remove excess surface moisture. The SAC was sieved to < 10 mm to remove coarse debris and obtain a uniform particle size; no chemical or thermal regeneration was applied so that its direct reuse potential could be evaluated. The raw materials were homogenised before mixing, and their moisture content, pH, and C/N ratio were determined before composting.

### Composting setup and operational conditions

Composting was conducted for 60 days under a roofed structure on a concrete base to minimise nitrogen leaching and external moisture intrusion (Zainudin et al. 2020). Four modified 90-L cylindrical bins provided passive aeration through three vertical tiers of 5-cm holes, ten additional holes, and an open base. CM (premixed with sawdust) and SMS were combined at a 2:1 dry-weight ratio. SAC was added at 0% (Control), 10% (T1), 20% (T2), and 30% (T3) (w/w). The mixtures were homogenised and turned every 3 days, and temperature was monitored at three depths. Composting units and physicochemical analyses were performed in duplicate. Given this level of replication, treatment differences were interpreted descriptively using convergent physicochemical, biological, and maturity indicators; no inferential claim of statistical significance was made.

### Composting performance monitoring

The impact of SAC dosage on composting performance was evaluated through key physicochemical and process indicators, including temperature, moisture content, pH, gas emissions, and time required to reach stabilisation. Moisture content was maintained within the optimal range of 30–60% through periodic water addition to support microbial activity. Compost temperature was monitored using a thermocouple inserted into the core region, serving as an indicator of microbial metabolic intensity.

Compost pH was determined using the supernatant method described by Omar et al. (2021). Briefly, 2 g (dry weight) of compost was mixed with 20 mL of distilled water (1:10 w/v), stirred at 300 rpm for 1 h, and centrifuged at 3,500 rpm for 15 min; the supernatant pH was then measured with a calibrated probe. Moisture content was determined using a moisture analyser (MX/MF-50, USA) with a 3-g sample. Odour was monitored qualitatively as a supporting maturity indicator. A portable multigas analyser (S360, Henan Zhong An, China) was used to measure oxygen (O₂) and carbon dioxide (CO₂) concentrations as indicators of aerobic respiration and decomposition intensity.

### Final compost quality and maturity assessment

The final composts were evaluated for nutrient composition, maturity, and phytotoxicity. Before nutrient analysis, samples were oven-dried at 60 °C to constant weight, ground, and homogenised. Total carbon (C), nitrogen (N), hydrogen (H), and sulfur (S) were determined using a LECO TruMac CNS elemental analyser (LECO Corporation, MI, USA). Dried samples were acid-digested for macro- and micronutrient quantification by inductively coupled plasma optical emission spectrometry (ICP-OES; PerkinElmer, USA). The elements analysed were phosphorus (P), potassium (K), calcium (Ca), magnesium (Mg), iron (Fe), zinc (Zn), copper (Cu), and manganese (Mn). All results were reported on a dry-weight basis.

Compost maturity was assessed using a combination of carbon-to-nitrogen (C/N) ratio and Solvita maturity index, which integrates CO₂ respiration and NH₃ emission profiles. Compost was considered mature when the C/N ratio was below 20 and the Solvita Index reached ≥ 7 (Norrrahim et al. 2022).

Phytotoxicity was evaluated using the germination index (GI) following the Thai Agricultural Standard (TAS, 2005). Compost extracts were prepared at 1:20 (w/v) and applied to green bean (*Vigna radiata* L.) seeds placed on Whatman Grade 1 filter paper, with 10 seeds per plate and three replicate plates. Each plate received 5 mL of extract and was incubated in the dark at 28–30 °C for 72 h; distilled water was used as the control. Germinated seeds and mean root length were recorded, and GI was calculated using Eq. (1), where GS denotes the number of germinated seeds and RL denotes mean root length (cm) for the compost extract and water control.

1 $$\:GI\%=\frac{{GS}_{compost}\times\:{RL}_{compost}}{{GS}_{control}\times\:{RL}_{control}}$$

### 16 S rRNA amplicon sequencing and bacterial community analysis

Compost (1 g) was collected on Days 0, 6, 36, and 60, corresponding to the mesophilic, thermophilic, cooling, and maturation sampling points, respectively. DNA was extracted and purified using the NucleoSpin^®^ Soil DNA Extraction Kit (Macherey-Nagel, Germany) according to the manufacturer’s protocol. DNA quality and quantity were assessed by NanoDrop spectrophotometry and agarose-gel electrophoresis. The V3–V4 region of the 16 S rRNA gene was amplified for 25 cycles using KAPA HiFi HotStart ReadyMix (Kapa Biosystems, Wilmington, MA, USA) with primers 341 F (5′-CCTACGGGNGGCWGCAG-3′) and 785R (5′-GACTACHVGGGTATCTAATCC-3′). Amplicons were purified using AMPure XP beads, tagged with dual indices (Nextera XT Index Kit), normalised, pooled, and sequenced on an Illumina MiSeq platform using 301, 8, 8, and 301 cycles for the forward read, Index 1, Index 2, and reverse read, respectively. Reads were demultiplexed, denoised, clustered, and assembled into operational taxonomic unit (OTU) tables in QIIME 2, and taxonomy was assigned using the SILVA 138 SSU database (Zainudin et al. 2020). Observed OTUs, Chao1 richness, Shannon diversity, and Faith’s phylogenetic diversity (Faith PD) were calculated in QIIME 2. The mean post-filtering sequencing depth was approximately 16,000 reads per sample. Given the duplicate composting design, community-composition and diversity differences were interpreted descriptively, without inferential p-values. Raw sequencing data are available from the corresponding author upon reasonable request.

## Results and discussion

### Physicochemical characteristics of raw materials

The physicochemical parameters of the raw materials (Table 1) exhibited complementary attributes conducive to co-composting. Chicken manure (CM) exhibited a low carbon-to-nitrogen (C/N) ratio of 11.77 and a high nitrogen concentration of 2.99%, thereby affirming its function as a nitrogen-dense substrate that effectively enhances microbial activity. Poultry manure generally has a C/N ratio of 8–15 and a nitrogen concentration of 2–5%, facilitating fast biodegradation but heightening the risk of ammonia volatilisation when the C/N ratio falls below 15 (Bernal et al. 2009). Ammonia emissions can result in nitrogen loss exceeding 90% if inadequate carbon-rich materials are integrated (Shan et al. 2021).

**Table 1:** Physicochemical characteristics of raw materials used in the co-composting system, including chicken manure (CM), spent mushroom substrate (SMS), and spent activated carbon (SAC)

Parameter	CM	SMS	SAC
pH	7.82	5.84	6.99
Moisture (%)	32.46	58.19	33.78
Carbon, C (%)	35.19	43.97	67.56
Nitrogen, N (%)	2.99	0.83	0.25
Hydrogen, H (%)	5.766	6.084	2.745
Sulfur, S (%)	0.444	0.101	0.228
C/N ratio	11.77	52.98	270.24
Phosphorus, P (%)	0.13	0.7	3.02
Potassium, K (%)	0.03	0.65	4.17
Copper, Cu (mg/L)	21.38	6.69	102.64
Manganese, Mn (mg/L)	83.6	80.3	649.60
Zinc, Zn (mg/L)	9.05	41.3	543.40
Lead, Pb (mg/L)	9.25	4.93	2.24
Aluminium, Al (mg/L)	3574.2	29.521	699.64

SMS had a high C/N ratio (52.98), consistent with its lignocellulosic composition and its role as a carbon-rich bulking agent. The C/N ratio of SMS varies widely (approximately 15–100) with substrate composition, while its cellulose, hemicellulose, and lignin can improve compost structure, aeration, and moisture distribution (Ma et al. 2025). Carbon-rich materials are commonly used to approach an initial composting C/N ratio of approximately 25–30, which supports microbial decomposition (Haug 1993; Huzir et al. 2026).

SAC had a high C/N ratio (270.24) and carbon content (67.56%), consistent with a stable, recalcitrant carbon matrix. Its porous structure may serve as a physical amendment and provide bacterial attachment sites, while studies of related carbonaceous amendments have reported reduced ammonia and methane emissions (Awasthi et al. 2020; Chen et al. 2017). The SAC pH was near neutral (6.99), whereas its moisture content (33.78%) was below the commonly recommended composting range of 40–60%, supporting the need for moisture adjustment during operation (Bernal et al. 2009).

SMS supplied phosphorus (0.70%) and potassium (0.65%), whereas SAC contained higher concentrations of phosphorus (3.02%) and potassium (4.17%), potentially reflecting mineral accumulation during its previous adsorption service (Kizito et al. 2019; Leong et al. 2022). SAC also contained higher Cu (102.64 mg/L), Mn (649.60 mg/L), and Zn (543.40 mg/L) than CM and SMS, consistent with the capacity of exhausted adsorbents to retain metals (Inyang et al. 2016). Aluminium was highest in CM (3,574.2 mg/L), while Pb remained low in all raw materials. Thus, CM provided readily available nitrogen, SMS supplied biodegradable carbon and structural support, and SAC contributed a recalcitrant carbon matrix. Although trace-metal bioavailability was not measured, the bacterial succession described in Sect. Bacterial community composition, structure, and succession and the absence of phytotoxicity in the final compost indicate that no detectable inhibition occurred under the conditions tested.

### Composting performance

**Fig. 1:**
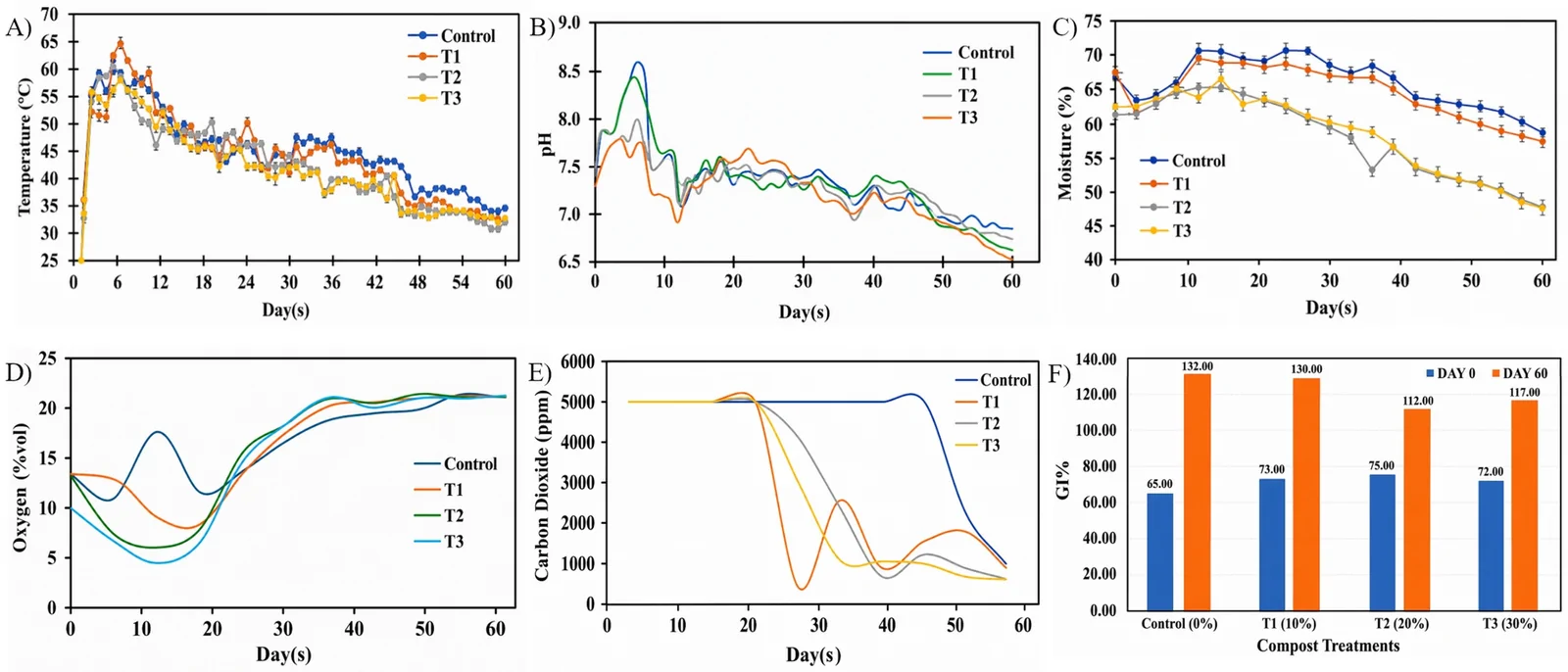
(**A**) Temperature, (**B**) pH, and (**C**) moisture profiles during composting for the Control (0% SAC), T1 (10% SAC), T2 (20% SAC), and T3 (30% SAC). (**D**) O₂ and (E) CO₂ concentration profiles across treatments. (**F**) Germination indices during the initial mesophilic and final maturation stages

Composting performance was evaluated from temperature, pH, and moisture profiles (Fig. 1). All treatments entered the thermophilic phase rapidly and exceeded 50 °C within the first 5 days, indicating active degradation of readily available substrates. The Control and T1 remained thermophilic for 12 days, whereas T2 and T3 remained thermophilic for 9 days, a 25% shorter duration (Table 2). Peak temperatures were 58.67 °C (Control), 63.67 °C (T1), 60.33 °C (T2), and 57.33 °C (T3). The higher T1 peak is consistent with vigorous microbial respiration, whereas the shorter phase at higher SAC doses may also reflect dilution of biodegradable substrate; therefore, thermophilic duration alone does not demonstrate microbial enhancement. Nevertheless, every treatment remained above 55 °C for at least three consecutive days, meeting the cited pathogen-inactivation criterion (Xie et al. 2025; Zainudin et al. 2020). When considered together with the earlier return towards ambient temperature, declining late-stage CO₂, and Solvita indices, the profiles support faster operational stabilisation in the SAC-amended systems.

Compost pH increased during the thermophilic phase as ammonification and nitrogen mineralisation proceeded (Fig. 1B) (Chung et al. 2023). Peak pH reached 8.4–8.5 in the Control and T1 but remained at 7.8–8.0 in T2 and T3. These lower peaks are consistent with buffering and possible NH₄⁺ adsorption by SAC, although adsorption was not measured directly. By Day 60, all treatments had converged to near-neutral pH values of 6.5–6.9, consistent with mature compost. The weaker alkalisation than is often observed with fresh biochar may reflect SAC surface modification during its previous adsorption service (Stacey et al. 2024; Zainudin et al. 2020).

Moisture increased after water was added on Day 12 and then declined through evaporation and microbial activity (Fig. 1C) (Ghanney et al. 2023). From Day 15, the Control and T1 retained approximately 59–60% moisture, whereas T2 and T3 reached final values near 48%. This greater moisture decline may reflect altered pore structure and gas exchange in the SAC-amended mixtures, although air-filled porosity was not measured (Jain et al. 2018). Overall, SAC dosage changed composting dynamics: T1 showed the highest peak temperature, while T2 and T3 had shorter thermophilic periods and earlier cooling. Together with the gas and maturity indicators, these associations support a shorter active composting period rather than a uniform increase in microbial activity at every SAC dosage.

**Table 2:** Duration of composting stages in the control and SAC-amended treatments

Treatment	Mesophilic (days)	Thermophilic (days)	Cooling phase (days)	Maximum temperature (°C)
Control (0% SAC)	1	12	46	58.67
T1 (10% SAC)	1	12	46	63.67
T2 (20% SAC)	1	9	49	60.33
T3 (30% SAC)	1	9	49	57.33

### Oxygen and carbon dioxide profiles during composting

The oxygen profiles (Fig. 1D) reflect the balance between oxygen supply and microbial consumption. All SAC-amended treatments showed rapid initial O₂ depletion, most notably T2 and T3; T3 reached approximately 4.5% (v/v) between Days 12 and 20. These low concentrations indicate high oxygen demand and do not, by themselves, demonstrate improved aeration. Because all bins used the same passive-aeration design, the early depletion is consistent with more intense respiration, but bulk density and air-filled porosity were not measured (Ma et al. 2022; Stacey et al. 2024). The Control temporarily increased to approximately 17% (v/v) before declining. After Day 25, O₂ progressively returned towards ambient levels as readily biodegradable substrates were depleted and respiration diminished, indicating transition towards stabilisation.

The CO₂ profiles (Fig. 1E) reflected microbial respiration and organic-carbon mineralisation (Wang et al. 2014). All treatments reached approximately 5,000 ppm during the thermophilic phase. The Control maintained elevated CO₂ until approximately Day 48, whereas the SAC-amended treatments declined earlier, particularly T1 after Day 20. This earlier decline is consistent with earlier depletion of readily degradable carbon and a shorter active bio-oxidative phase. Direct CO₂ adsorption and microbial carbon-use efficiency were not measured, so the mechanism cannot be assigned from the gas profile alone. Similar late-stage CO₂ reductions have been reported for composts containing carbonaceous amendments (Stegenta-Dąbrowska et al. 2024; Yan et al. 2022).

### Compost maturity

In this study, operational stabilisation was defined by near-ambient temperature, reduced respiratory activity (Solvita CO₂/NH₃ scores ≥ 6), and declining gas emissions, whereas maturity additionally required a GI above 80%. The Solvita results (Table 3) documented progression in stability. At Day 0, all treatments recorded CO₂/NH₃ scores of 2/3, consistent with immature compost. At Day 5, CO₂ scores ranged from 3 in the Control to 5 in T2, indicating active respiration, while the low NH₃ scores were consistent with substantial ammonia volatilisation during rapid nitrogen mineralisation.

By Day 30, CO₂/NH₃ scores had increased to 6/5–6/6 across treatments, indicating reduced respiration and transition towards stabilisation. T1 recorded the highest NH₃ score (6) at Days 30 and 60, consistent with the lowest residual ammonia, whereas T2 and T3 reached the highest CO₂ score (7) at Day 60, consistent with the lowest residual respiration. Thus, different SAC doses affected the two gaseous maturity indicators differently. When the Solvita scores were considered together with temperature and continuous gas profiles, the SAC-amended systems reached operational stabilisation within 30–42 days, and all treatments were mature by Day 60. Lower NH₃ emissions are consistent with moderated volatilisation, but nitrogen retention cannot be quantified without a nitrogen mass balance.

**Table 3:** Solvita CO₂/NH₃ scores derived from the Solvita colour scale on Days 0, 5, 30, and 60

Day	Control (CO₂/NH₃)	T1 (CO₂/NH₃)	T2 (CO₂/NH₃)	T3 (CO₂/NH₃)	Maturity status
0	2/3	2/3	2/3	2/3	Immature
5	3/1	4/1	5/1	4/1	Immature
30	6/5	6/6	6/5	6/5	All treatments: stabilising to near-mature
60	6/5	6/6	7/5	7/5	Mature; T2 and T3 had the highest CO₂ scores

The comparison in Table 4 shows that conventional systems commonly require 60–120 days to reach maturity, while the cited biochar-amended system required 56–70 days (Khan et al. 2014). In the present study, operational stabilisation was observed within 30–42 days from near-ambient temperature, declining gas emissions, and Solvita CO₂/NH₃ indices (Fig. 1D–E). Stabilisation and maturity are not equivalent: stabilisation denotes reduced biological activity, whereas maturity additionally requires the absence of phytotoxicity and suitability for plant growth. Accordingly, full maturity was confirmed at Day 60 by GI values above 100%. Cross-study durations should be interpreted cautiously because feedstock, scale, aeration, and maturity criteria differ among systems.

**Table 4:** Comparison of compost stabilisation or maturity time with previous studies

Feedstock	Additive	System	Time to stabilisation/maturity (days)	Maturity indicator	Reference
Chicken manure and SMS	SAC (10–30%)	In-vessel bin	30–42	Temperature, Solvita CO₂/NH₃, and GI	This study
Agri-food waste	Controlled system	Reactor	7–10	GI, N dynamics	(Gebremikael et al. 2022)
Chicken manure and sawdust	Biochar	Lab-scale pile	56–70	C/N, GI	(Khan et al. 2014)
Tomato residues and chicken manure	None	Rotary drum	60	GI, C/N	(Rashwan et al. 2021)
Goat manure and shell waste	None	Windrow	60–76	GI, C/N	(Zhang et al. 2019)
Mixed organic waste	None	Conventional	60–120	C/N, stability	(Lalremruati and Devi 2021)
Food waste	None	Composting	> 50	GI	(Joshi et al. 2010)

### Compost toxicity

Phytotoxicity was evaluated using the GI, which integrates seed germination and root elongation responses to soluble compounds. GI increased from the initial mesophilic stage to maturation (Fig. 1F), indicating progressive detoxification of the compost extracts. Initial values of approximately 60–80% were consistent with moderate inhibition by organic acids, ammonia, or other intermediates (Lee et al. 2020; Oviedo-Ocaña et al. 2025). At Day 60, every treatment exceeded 100%, indicating that none of the final extracts was phytotoxic and that germination and root growth exceeded the water control.

The Control and T1 had the highest GI values (132.03% and 129.87%, respectively), followed by T3 (117.39%) and T2 (111.48%). Values above 100% can occur when humic substances or microbial metabolites stimulate germination and root elongation (Lončarić et al. 2024). Because GI did not increase with SAC dosage, the assay does not support a dose-dependent improvement in agronomic response. The lower values at higher SAC doses may reflect nutrient dilution or residual adsorbates, but these mechanisms were not tested.

The GI values obtained in the present study were comparable to or higher than those reported in previous co-composting studies employing carbonaceous and lignocellulosic amendments. For example, Khan et al. (2014) reported GI values exceeding 80% in chicken manure compost amended with biochar after 56–70 days of composting, while Rashwan et al. (2021) observed mature compost GI values above 80% during co-composting of tomato residues and chicken manure. Similarly, Zeng et al. (2023) demonstrated that the incorporation of spent mushroom substrate improved compost phytotoxicity by enhancing humification and reducing inhibitory intermediates. The consistently high GI values (> 100%) observed in all SAC-amended treatments indicate effective detoxification and suggest that SAC incorporation did not induce phytotoxic effects under the conditions employed.

### Structural characterisation (FTIR and SEM–EDX analyses)

**Fig. 2:**
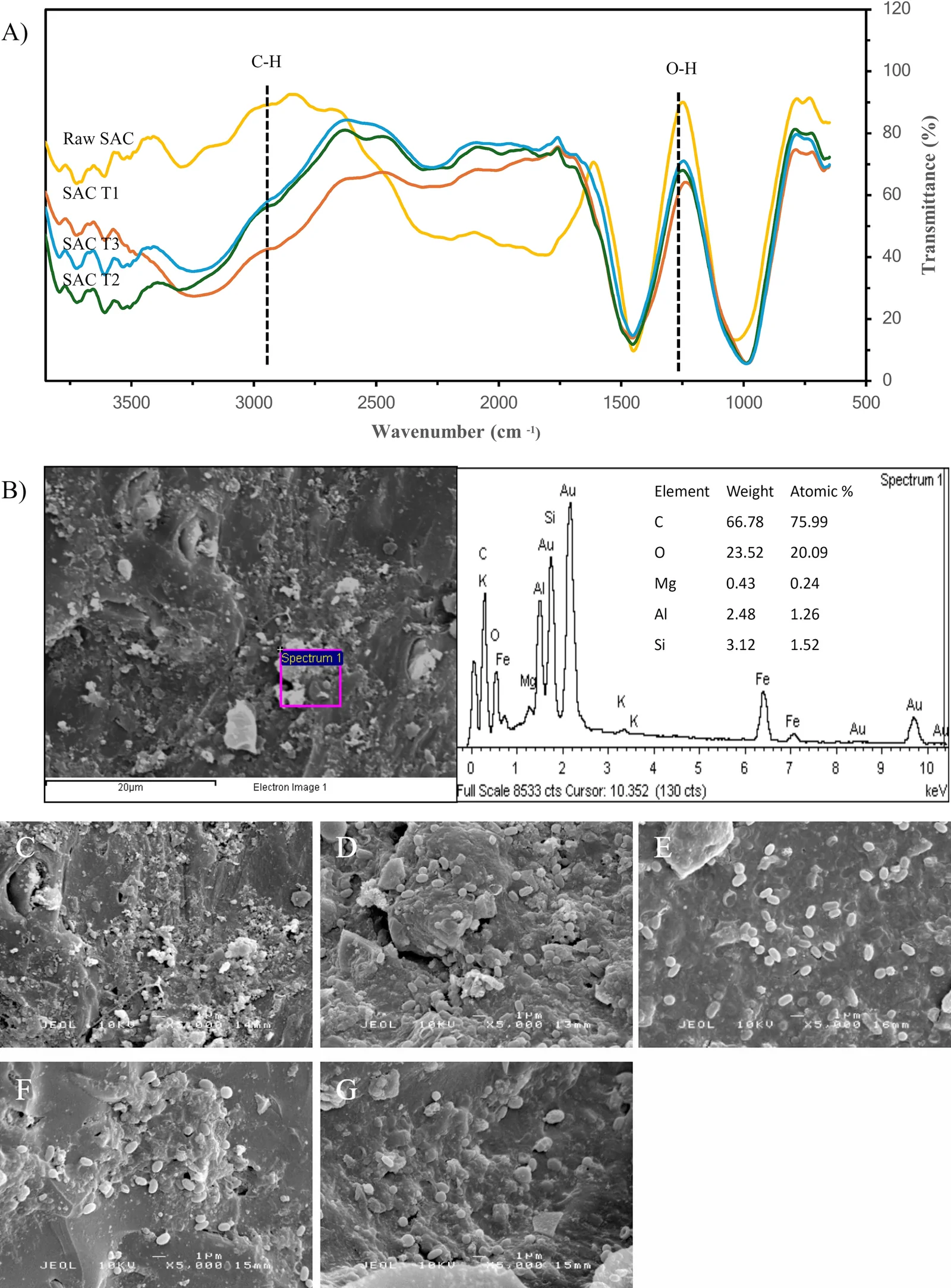
(**A**) FTIR spectra of raw SAC and mature SAC-amended composts: T1 (10%), T2 (20%), and T3 (30%). (**B**) SEM–EDX spectrum of raw SAC before composting. (**C**–**G**) SEM micrographs (×5,000) showing bacterial colonisation in (**C**) raw SAC, (**D**) Control, (**E**) T1, (**F**) T2, and (**G**) T3

The structural evolution of spent activated carbon (SAC) during composting was analysed using FTIR (Fig. 2A). Raw SAC exhibited characteristic bands at ~ 3200–3600 cm⁻¹ (O–H), ~ 2900 cm⁻¹ (C–H), and ~ 1500–1600 cm⁻¹ (aromatic C = C), confirming oxygen-containing functional groups on a carbonaceous structure, consistent with previous studies on activated carbon materials (Inyang et al. 2016; Keiluweit et al. 2010). After composting, reduced O–H and C–H peak intensities suggested microbial degradation of labile functional groups (Chefetz et al. 1996), while enhancement at ~ 1200–1000 cm⁻¹ indicated accumulation of polysaccharides and humic substances derived from microbial activity (Awasthi et al. 2017; Jindo et al. 2012). The stable aromatic C = C peak confirmed retention of the recalcitrant carbon backbone.

SEM–EDX analysis (Fig. 2B) showed that SAC consisted mainly of carbon (66.78 wt%) and oxygen (23.52 wt%), with smaller amounts of Si, Al, and Mg retained from previous use (Inyang et al. 2016). Raw SAC had a dense, irregular surface with limited visible pore openings, consistent with partial pore blockage (Fig. 2C). After composting, microbial cells and biofilm-like structures were visible on SAC surfaces. These observations support the role of SAC as a potential attachment matrix, although SEM images do not quantify microbial abundance or resolve microporosity (Awasthi et al. 2017; Lehmann et al. 2011).

The Control (Fig. 2D) showed dense colonies with rod-shaped and coccoid morphologies in compact biofilm-like structures. In T1 (Fig. 2E), cells were observed directly on SAC surfaces with a more dispersed distribution. T2 and T3 (Fig. 2F–G) showed increasingly dispersed colonies, particularly T3, which may reflect dilution of biodegradable substrate at higher SAC doses. This descriptive pattern parallels the community results in Sect. Bacterial community composition, structure, and succession : T2 had the highest combined abundance of taxa associated in the literature with plant growth-promoting functions, whereas T3 had lower observed OTUs, Chao1 richness, and Shannon diversity than T1 and the Control at maturity. SEM morphology alone, however, cannot establish differences in bacterial abundance.

Taken together, the SEM observations and process profiles support SAC as a potential microbial carrier and structural modifier. SAC-amended systems showed a shorter high-activity decomposition phase and operational stabilisation within 30–42 days, approximately 30–50% faster than the literature comparators in Table 4. Improved aeration, adsorption, and microbial attachment provide plausible explanations, but these mechanisms were not quantified directly in the present experiment.

### Quality of the final compost product

The physicochemical properties of the final composts are summarised in Table 5. Final pH ranged from 6.53 to 6.83, within the cited ranges for agronomic use. Moisture in the Control and T1 remained above the cited commercial standards, indicating that additional drying would be required before storage or marketing rather than demonstrating biological immaturity. T2 and T3 had lower final moisture contents (48.24% and 47.74%), which may facilitate handling but still exceeded the listed national thresholds.

Total organic carbon increased with SAC dosage from 37.94% in the Control to 54.12% in T3, whereas total nitrogen concentration decreased from 2.28% to 1.34%, increasing the C/N ratio. The Control (16.64) met the operational maturity criterion of < 20, T1 (21.50) was slightly above that criterion but below the Malaysian limit of 25, and T2 (35.34) and T3 (40.39) exceeded both values (Shen et al. 2024). Nevertheless, near-ambient temperatures, favourable Solvita indices, and GI values above 100% indicated maturity and absence of phytotoxicity in all treatments. In SAC-amended systems, the high C/N ratio therefore reflects substantial recalcitrant carbon and should not be used as the sole indicator of biological maturity.

Humic acid varied from 2.25% to 3.25%, with T3 recording the highest value; this pattern may reflect humification on SAC surfaces, although temporal humic-acid formation was not measured. Nitrogen, phosphorus, and potassium concentrations generally decreased as SAC dosage increased, consistent with dilution by carbon-rich material. Cu ranged from 3.09 mg/kg in the Control to 17.86 mg/kg in T1. The origin of the higher T1 value was not resolved, although copper-containing feed additives can contribute Cu to poultry manure (Zheng et al. 2022). Pb remained low (2.55–6.97 mg/kg), well below the Malaysian limit of 300 mg/kg, while Mn and Al generally increased with SAC addition (Bernal et al. 2009; Zheng et al. 2022).

The declines in nitrogen, phosphorus, potassium, and GI with increasing SAC dosage show a trade-off between carbon enrichment and nutrient concentration. Some final properties did not meet every cited national specification: moisture exceeded the listed limits in all treatments, and nitrogen in T2 and T3 was below the Malaysian minimum of 1.5%, although it remained above the Thai minimum of 1.0%. GI values above 100% indicated no phytotoxicity, but additional drying or formulation may be required for compliance in specific markets.

Compost yield increased from 41.94% in the Control to 54.70% in T3, consistent with the increasing fraction of recalcitrant SAC. GI did not follow the same trend. T1 retained higher nitrogen and GI, T2 combined lower moisture with the highest abundance of taxa previously associated with plant growth-promoting functions, and T3 had the highest carbon content and yield but the strongest nutrient dilution. The preferred SAC dosage therefore depends on whether nutrient concentration, microbial attributes, or mass yield is prioritised.

**Fig. 3:**
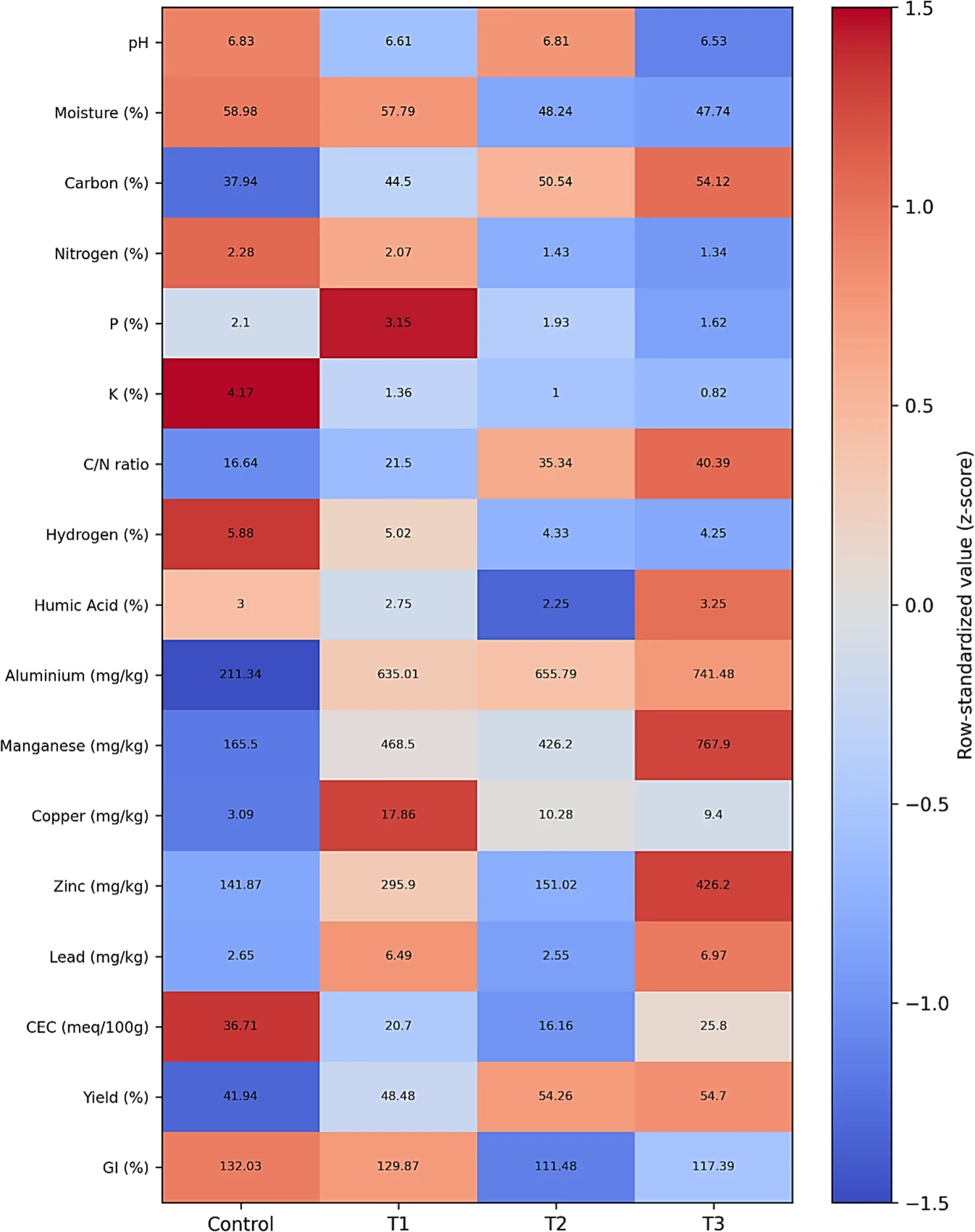
Integrated physicochemical heatmap of mature compost properties for the Control (0% SAC), T1 (10% SAC), T2 (20% SAC), and T3 (30% SAC), using row-standardised z-scores

The integrated heatmap (Fig. 3) summarises the dosage-dependent physicochemical patterns. Total organic carbon and compost yield increased from the Control to T3, while total nitrogen decreased and the C/N ratio increased. These are concentration and yield measurements; in the absence of an elemental mass balance, they should not be interpreted as direct measurements of carbon retention or nitrogen loss. The patterns are consistent with dilution by carbon-rich, recalcitrant SAC (Awasthi et al. 2020).

Despite the elevated C/N ratios in T2 and T3, every treatment had a GI above 100%, indicating absence of phytotoxicity and supporting successful maturation. This confirms that C/N ratio alone does not fully represent biological maturity when a compost contains substantial recalcitrant carbon (Bernal et al. 2009). The heatmap also shows higher Al, Mn, and Zn at some SAC dosages, consistent with inorganic residues retained during previous adsorption service (Inyang et al. 2016). Pb remained well below the cited regulatory limits.

T1 retained the strongest nutrient and GI profile among the SAC treatments, whereas T2 combined lower moisture with the highest abundance of taxa associated with plant growth-promoting functions. T3 maximised carbon content and yield but had lower nutrient concentrations. These contrasting profiles show that SAC dosage should be optimised against the intended compost quality and use rather than judged from a single maturity indicator.

**Table 5:** Comparison of the physicochemical characteristics of the final composts with organic-fertiliser standards in selected ASEAN countries

Parameter	Treatment	T1	T2	T3	Malaysia^a^	Thailand^b^	Indonesia^c^
	Control						
pH	6.83	6.61	6.81	6.53	5.0–8.0	5.5–8.5	4.0–9.0
Moisture (%)	58.98	57.79	48.24	47.74	< 30%	≤ 35%	15–25%
Carbon, C (%)	37.94	44.5	50.54	54.12	NR	NR	NR
Nitrogen, N (%)	2.28	2.07	1.43	1.34	> 1.5%	≥ 1.0%	N + P_2_O_5_ + K_2_O > 4%
Phosphorus, P (%)	2.10	3.15	1.93	1.62	NR	≥ 0.5%	
Potassium, K (%)	4.17	1.36	1.00	0.82	NR	≥ 0.5%	
C/N ratio	16.64	21.5	35.34	40.39	< 25:1	< 20:1	15:1–25:1
Hydrogen, H (%)	5.88	5.02	4.33	4.25	NR	NR	NR
Humic acid (%)	3.0	2.75	2.25	3.25	NR	NR	NR
Aluminium, Al (mg/kg)	211.34	635.01	655.79	741.48	NR	NR	NR
Manganese, Mn (mg/kg)	165.5	468.5	426.2	767.9	NR	NR	NR
Copper, Cu (mg/kg)	3.09	17.86	10.28	9.40	NR	≤ 500 mg/kg	NR
Zinc, Zn (mg/kg)	141.87	295.9	151.02	426.2	NR	NR	< 5000 mg/kg
Lead, Pb (mg/kg)	2.65	6.49	2.55	6.97	< 300 mg/kg	≤ 500 mg/kg	< 50 mg/kg
CEC (meq/100 g)	36.71	20.70	16.16	25.8	NR	NR	NR
Compost yield (%)	41.94	48.48	54.26	54.7	NR	NR	NR
GI (%)	132.03	129.87	111.48	117.39	NR	NR	NR

^a^Malaysia Standard for Organic Fertilisers MS 1517:2012 (2012) (Organic Fertilisers Specification)

^b^Thai Agricultural Standard. TAS 9503. 2005

^c^Ministry of Agriculture Republic of Indonesia (2011) Minimum Technical Requirements for Organic Fertilizer

^*^NR parameter not specified in national compost standards

### Bacterial community composition, structure, and succession

#### Alpha diversity and bacterial richness

Because the composting experiment was performed in duplicate, alpha-diversity metrics were interpreted descriptively (Table 6). From Day 0 to Day 60, observed OTUs, Chao1 richness, and Faith PD increased in every treatment, while Shannon diversity generally increased after the thermophilic sampling point. At Day 60, T1 had the highest observed OTUs (197), Chao1 richness (213), and Faith PD (23.14), compared with 188, 211, and 20.24 in the Control. T2 and T3 had fewer observed OTUs and lower Chao1 richness than the Control but higher Faith PD, while the Control had the highest Shannon diversity (6.94). SAC therefore did not increase every diversity metric uniformly. T1 most consistently increased richness and phylogenetic breadth, whereas T2 and T3 produced more selective shifts in community structure.

**Table 6:** Bacterial alpha-diversity metrics across treatments and composting stages

Treatment	Day	Observed OTUs	Chao1	Shannon	Faith PD
Control (0% SAC)	0	37	51	5.99	4.01
	6	81	95	5.77	12.28
	36	160	187	6.63	19.52
	60	188	211	6.94	20.24
T1 (10% SAC)	0	67	80	5.63	7.33
	6	101	119	5.78	12.96
	36	149	160	6.50	19.09
	60	197	213	6.83	23.14
T2 (20% SAC)	0	51	61	5.26	6.72
	6	80	88	5.30	11.62
	36	136	152	6.35	17.95
	60	179	205	6.88	21.87
T3 (30% SAC)	0	59	76	4.99	8.04
	6	80	90.25	5.65	11.06
	36	159	180	6.70	18.33
	60	186	200	6.74	21.39

#### Phylum- and genus-level bacterial community structure

**Fig. 4:**
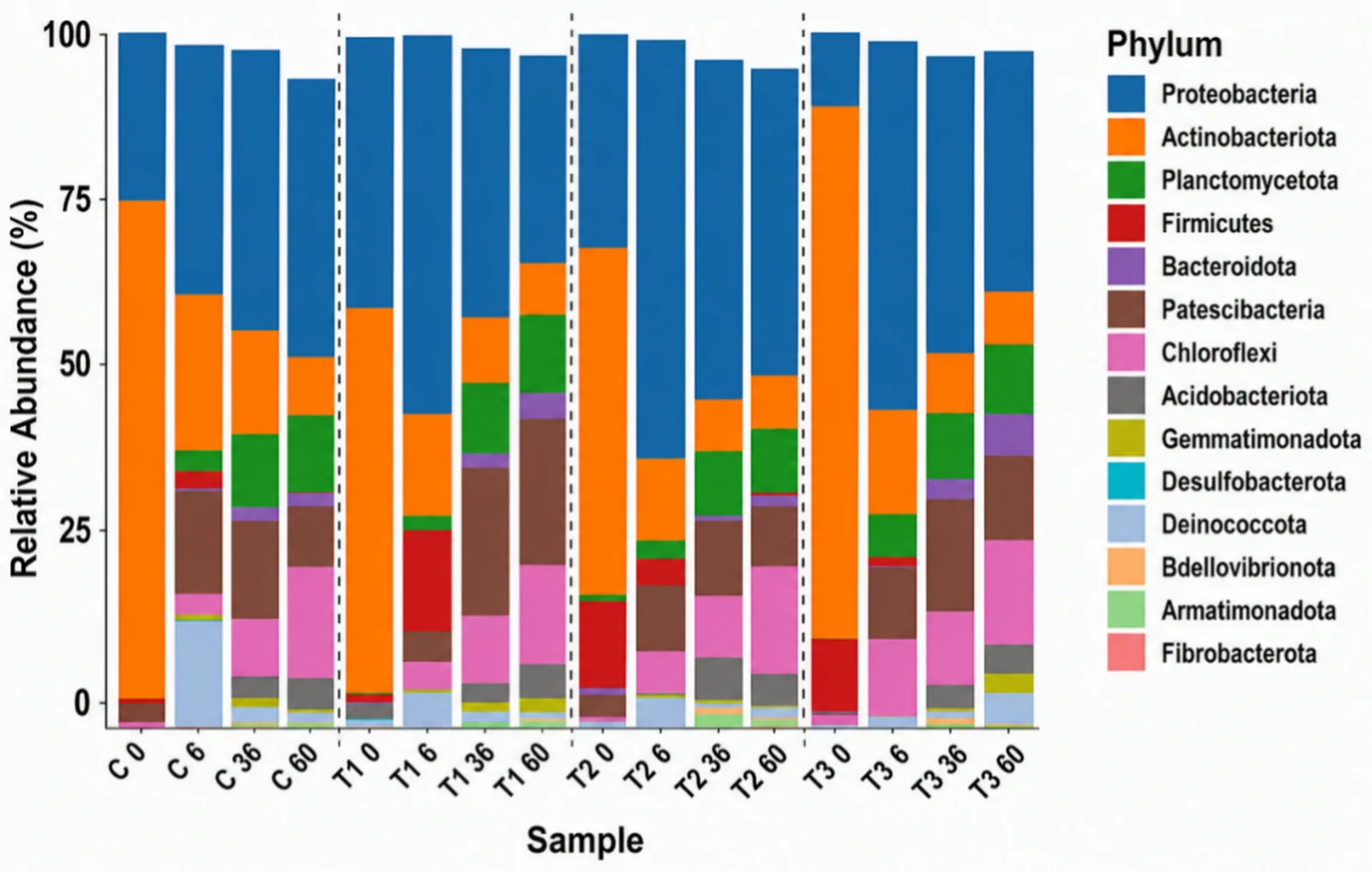
Relative abundance of the 14 dominant bacterial phyla at the mesophilic, thermophilic, cooling, and maturation sampling points. Low-abundance phyla and reads outside the displayed categories were omitted for visual clarity; therefore, some stacked bars total less than 100%

Figure 4 displays the 14 dominant bacterial phyla. Because only these displayed categories were plotted, low-abundance phyla and reads outside the displayed categories were omitted for clarity; consequently, some stacked bars total less than 100%. Proteobacteria and Actinobacteriota dominated in a treatment- and stage-dependent manner. At Day 0, Actinobacteriota was prominent, particularly in T3, consistent with the occurrence of taxa often associated with degradation of complex lignocellulosic substrates (Zeng et al. 2023). At Day 6, Proteobacteria increased markedly in T2 (60.81%) and T3 (53.63%), while Firmicutes also increased in T3, consistent with enrichment of thermotolerant and spore-forming groups during the thermophilic stage (Aguilar-Paredes et al. 2023; Lepesteur et al. 2025). Proteobacteria remained prominent at the cooling (Day 36) and maturation (Day 60) sampling points, indicating continued restructuring of the bacterial community. Relative abundance alone does not demonstrate metabolic activity; prolonged substrate availability and SAC microhabitats are proposed explanations rather than directly measured mechanisms.

The genus-level heatmap (Fig. S1) contains 28 dominant genera (> 0.5% relative abundance). An unclassified taxon within Rhizobiaceae was among the most abundant groups, and T2 had the highest combined relative abundance (22.47%) of taxa previously associated with plant growth-promoting functions. These taxa included *Mesorhizobium*, *Ensifer*, *Devosia*, *Sphingopyxis*, and *Altererythrobacter* (Fahde et al. 2023; Fanai et al. 2024). Their taxonomic detection does not demonstrate nitrogen fixation or plant growth promotion in this compost because functional genes and activities were not measured. *Brevibacterium* and *Brachybacterium* were prominent in early T3 samples; members of these genera have been linked to extracellular-enzyme production and organic-matter decomposition (Aguilar-Paredes et al. 2023). Overall, SAC caused selective, dosage-dependent restructuring of bacterial taxa rather than a uniform expansion of the entire community.

#### Bacterial succession across composting stages

**Fig. 5:**
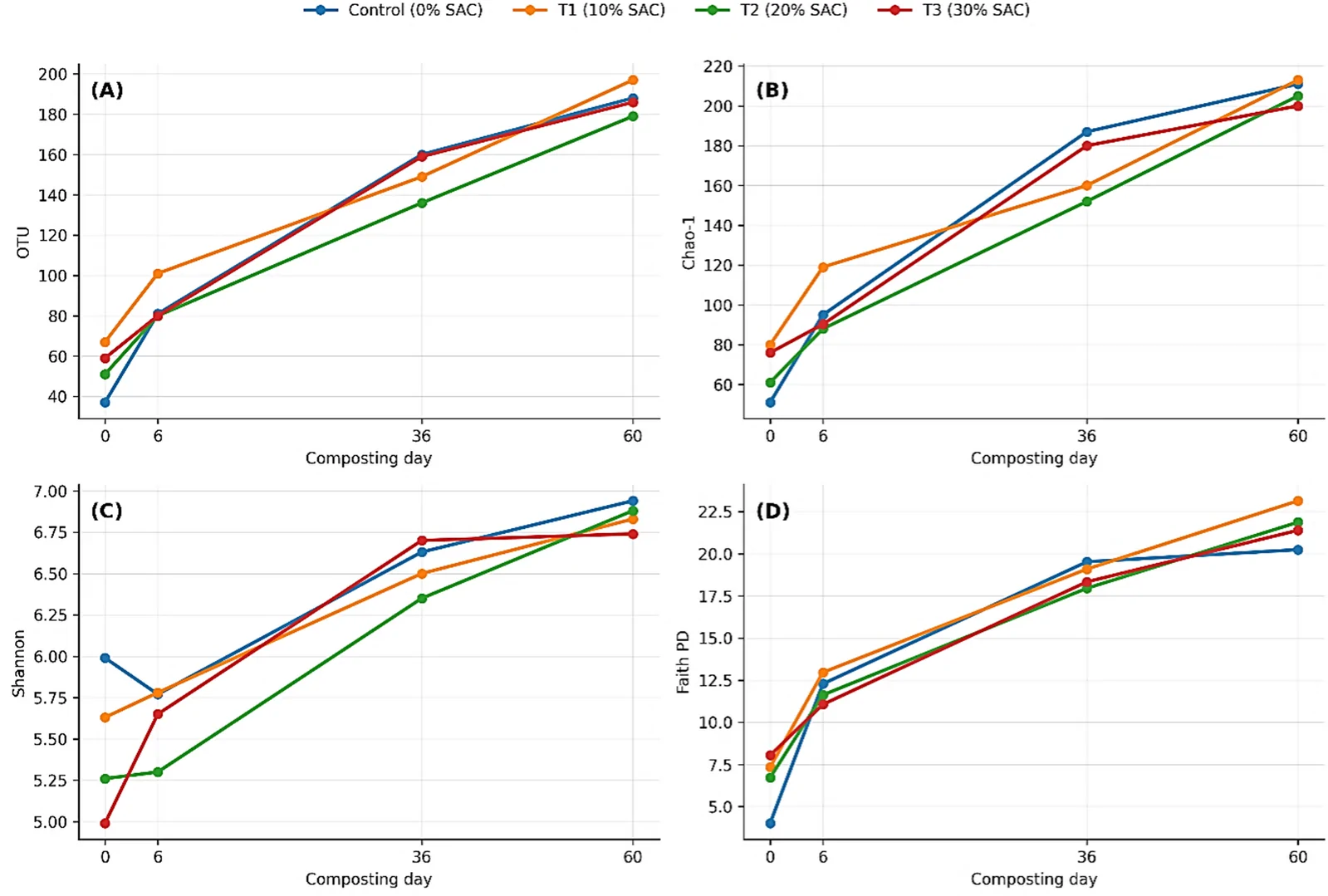
Temporal changes in bacterial alpha-diversity indices during SAC-amended composting: (**A**) observed OTUs, (**B**) Chao1 richness, (**C**) Shannon diversity, and (**D**) Faith’s phylogenetic diversity (Faith PD) for the Control (0% SAC), T1 (10% SAC), T2 (20% SAC), and T3 (30% SAC). Vertical dashed lines separate the stage-associated sampling points: Day 0 (mesophilic), Day 6 (thermophilic), Day 36 (cooling), and Day 60 (maturation). The lines are visual guides and do not represent treatment-specific temperature transition times, which varied as reported in Table 2

The stage-associated trajectories in Fig. 5 show succession from the mesophilic (Day 0) and thermophilic (Day 6) samples to cooling (Day 36) and maturation (Day 60). Observed OTUs and Chao1 richness increased substantially from the initial to the final sampling point in every treatment. T1 reached the highest mature values (197 OTUs and Chao1 = 213), followed by the Control for Chao1 (211) and observed OTUs (188). The temporal increase in richness and phylogenetic breadth coincided with the decline from high temperature and respiration to near-ambient conditions and lower gas emissions. This temporal concordance links bacterial succession with compost stabilisation, but it does not by itself establish causality. The development of additional niches during organic-matter transformation provides a plausible ecological explanation (Aguilar-Paredes et al. 2023).

At Day 60, Shannon diversity ranged from 6.74 to 6.94 (Fig. 5C), with the Control recording the highest value. All SAC-amended treatments were slightly lower, indicating that SAC did not uniformly enhance evenness and diversity. In contrast, Faith PD increased from 4.01 to 8.04 at Day 0 to 20.24–23.14 at Day 60 (Fig. 5D), and all SAC treatments exceeded the Control at maturity; T1 was highest (23.14). The combination of lower Shannon diversity and higher Faith PD indicates selective enrichment across a broader phylogenetic range rather than equal expansion of all taxa. Improved habitat heterogeneity on carbonaceous surfaces is a plausible mechanism, but it was not measured directly (Lehmann et al. 2011; Zainudin et al. 2020).

Richness increased between Days 0 and 6 in every treatment, showing rapid bacterial turnover during the thermophilic stage, and continued to increase through cooling and maturation. At Day 60, T3 had slightly fewer observed OTUs and lower Chao1 and Shannon values than the Control but a higher Faith PD, consistent with selective pressure at the highest SAC dosage. Overall, T1 produced the strongest increase in richness and phylogenetic breadth, whereas T2 produced the greatest enrichment of taxa previously associated with plant growth-promoting functions. Thus, moderate SAC promoted favourable bacterial succession through different community attributes rather than through a uniform increase in total diversity.

#### Relationships between bacterial communities and compost properties

**Fig. 6:**
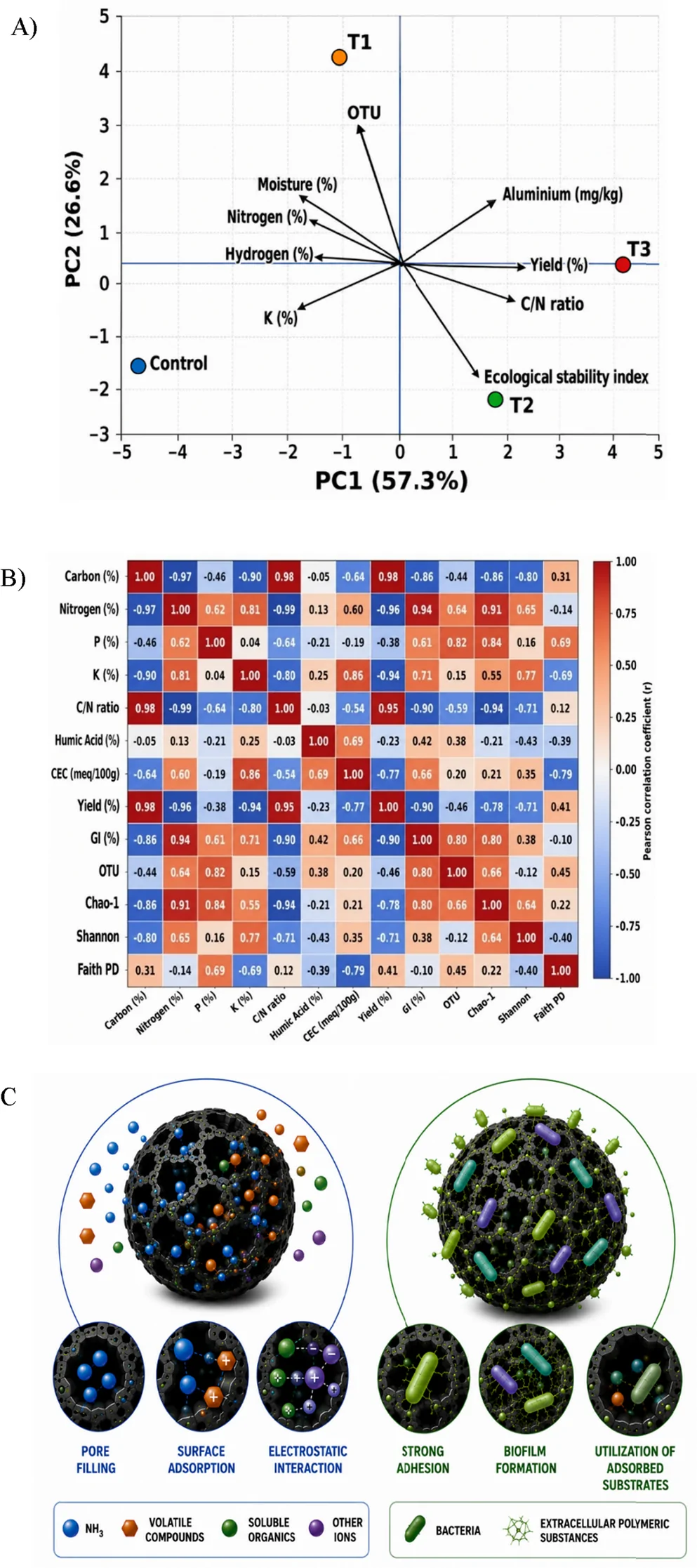
(**A**) PCA biplot integrating physicochemical and bacterial community properties of the mature composts; PC1 and PC2 explain 57.3% and 26.6% of the variance, respectively. (**B**) Exploratory Pearson correlation heatmap showing associations between physicochemical properties and bacterial alpha-diversity indices. (**C**) Conceptual model of the proposed roles of spent activated carbon (SAC) during co-composting

The Pearson correlation heatmap in Fig. 6B is exploratory because it is based on four treatment-level values at maturity; the coefficients describe co-variation and are not inferential evidence of causal relationships. Total organic carbon, compost yield, and C/N ratio co-varied positively and were negatively associated with nitrogen, potassium, and GI, consistent with dosage-dependent carbon enrichment and nutrient dilution. Observed OTUs and Chao1 richness were positively associated (*r* = 0.66), as were Chao1 and Shannon diversity (*r* = 0.64), whereas relationships involving Faith PD were mixed. GI was positively associated with nitrogen (*r* = 0.94), potassium (*r* = 0.71), observed OTUs (*r* = 0.80), and Chao1 richness (*r* = 0.80). CEC showed weak positive associations with observed OTUs, Chao1, and Shannon but a negative association with Faith PD; therefore, the data do not support a general claim that CEC increased all dimensions of bacterial diversity. Overall, the matrix indicates a descriptive trade-off between carbon enrichment, nutrient concentration, bacterial community attributes, and GI.

Figure 6C presents a conceptual model for the possible physicochemical and biological roles of SAC. The directly observed evidence comprises temperature and gas profiles, Solvita indices, SEM evidence of cell attachment, and treatment- and stage-dependent bacterial community shifts. Adsorption of NH₃, dissolved organic compounds, and volatile intermediates, together with provision of microbial attachment sites, offers a plausible link between SAC addition and the earlier operational stabilisation, but these mechanisms were not measured directly.

The porous structure and surface chemistry of carbonaceous materials can support adsorption and protected microbial microhabitats (Awasthi et al.^2017^; Lehmann et al. 2011). In this study, SEM directly showed cells attached to SAC surfaces, while sequencing showed selective enrichment of taxa previously associated with nitrogen cycling, organic-matter decomposition, and plant growth-promoting functions, including Rhizobiaceae, *Mesorhizobium*, and *Ensifer* at moderate SAC dosages. SAC addition was also associated with moderated pH, earlier decline in late-stage respiration, and favourable Solvita indices. Together, these observations support the interpretation that SAC addition alters the physicochemical habitat, selectively shapes bacterial succession, and consequently promotes earlier compost stabilisation. It remains a proposed mechanism rather than a demonstrated causal pathway because adsorption capacity, functional genes, and taxon-specific metabolic rates were not measured. At 30% SAC, substrate dilution may have increased selective pressure and limited broader richness.

Table 7 places the present microbial results in the context of other carbonaceous amendments. T2 did not maximise overall alpha diversity; instead, it had the highest combined abundance (22.47%) of taxa previously associated with plant growth-promoting functions, including Rhizobiaceae, *Mesorhizobium*, *Ensifer*, and *Devosia*. T1 had the highest observed richness and Faith PD. Thus, moderate SAC selectively restructured different bacterial community attributes rather than universally enhancing the entire community. Compared with reports of reduced ammonia and composting time (Wang et al. 2022) or improved GI (Zou et al. 2025), this study adds evidence of passive taxonomic enrichment without external inoculation alongside operational stabilisation within 30–42 days. The proposed plant growth-promoting functions remain literature-based because they were not verified by functional assays

**Table 7:** Comparison of bacterial community responses in SAC-amended compost with microbial effects reported for other carbonaceous amendments

System/feedstock	Amendment	Main bacterial/community response	Reference
Chicken manure with SMS	20% SAC (T2)	Highest combined relative abundance (22.47%) of taxa previously associated with plant growth-promoting functions; Rhizobiaceae prominent; *Mesorhizobium*, *Ensifer*, and *Devosia* detected	This study
	Control (0% SAC)	GI = 132.03%; lower combined abundance of the literature-associated taxa than T2	
Turfgrass soil establishment	Biochar (12.5 to 25 t per ha) and compost	• Increased enzyme activity (6 to 54%) • Increased respiration (bacteria 88 to 161%, fungi 346 to 426%)	(Azeem et al. 2020)
Spinach under Pb stress	Compost with biochar	• PGPR such as *Bacillus* and *Alcaligenes* increased nutrient uptake • IAA and ACC deaminase activity enhanced	(Zafar-Ul-Hye et al. 2020)
Soil	Biochar with PGPR	• Increased bacterial diversity • *Actinobacteria* increased • Higher Simpson index with PGPR	(Ren et al. 2020)
Food waste digestate compost	Activated carbon	• Ammonia reduced by 34% • Composting time reduced by approximately 50% • Humification increased	(Wang et al. 2022)
Biochar compost	Biochar with compost	• Germination index increased by 25.57% • Improved microbial habitat	(Zou et al. 2025)

## Conclusion

This study shows that SAC is a promising carbonaceous amendment for chicken manure–SMS co-composting. SAC addition was associated with a shorter thermophilic phase (12 days in the Control versus 9 days in T2 and T3) and operational stabilisation within 30–42 days, supported by concordant temperature, gas-emission, and Solvita indicators. All treatments were mature by Day 60 and had GI values above 100%. Because composting units were duplicated and treatment comparisons were descriptive, these results should be interpreted as consistent associations rather than inferential proof of significance.

Increasing SAC dosage raised measured total organic carbon concentration from 37.94% to 54.12% and compost yield from 41.94% to 54.70%, but it also diluted nitrogen, phosphorus, and potassium and reduced GI relative to the Control. Without an elemental mass balance, these measurements do not quantify absolute carbon retention or nitrogen conservation. The elevated C/N ratios in T2 and T3 likewise reflected recalcitrant SAC carbon and did not indicate phytotoxic immaturity when evaluated alongside temperature, Solvita, and GI.

The bacterial data do not show a uniform increase in the whole community. At maturity, T1 had the highest observed OTUs, Chao1 richness, and Faith PD, while T2 had the highest combined abundance of taxa previously associated with plant growth-promoting functions. Shannon diversity in all SAC treatments remained slightly below the Control. The evidence therefore supports favourable, selective bacterial succession rather than blanket diversity enhancement. Considering stabilisation, community attributes, and compost quality together, moderate SAC addition (10–20%) is preferable to 30%, although the optimal dose depends on the intended product specification.

This study highlights the potential of SAC as a sustainable amendment for enhancing composting performance while providing a circular and environmentally beneficial valorisation pathway for spent activated carbon generated from water treatment processes.

## Supplementary Information

Below is the link to the electronic supplementary material.

Supplementary Material 1 (DOCX 1.03 MB)

## Data Availability

No datasets were generated or analysed during the current study.
