# Supplementary material for: Valorisation of spent activated carbon for accelerated compost stabilisation and enrichment of plant growth-promoting bacterial taxa
Source: World J Microbiol Biotechnol. 2026 Aug 27;42(9):496. doi: 10.1007/s11274-026-05223-2 (PMC13521986; doi:10.1007/s11274-026-05223-2)
Supplement: Supplementary file 1 [file 11274_2026_5223_MOESM1_ESM.docx]

**SUPPLEMENTARY INFORMATION**

**Valorisation of Spent Activated Carbon for Accelerated Compost Stabilisation and Enrichment of Plant Growth-Promoting Bacterial Taxa**

Halimatul Aliyah Othman^1^, Mohd Hafif Samsudin^2^, Mohammed Abdillah Ahmad Farid^2^, Ahmad Muhaimin Roslan^1^, Toshinari Maeda^3^, Mohd Huzairi Mohd Zainudin^4^, Hendrix Yulis Setyawan^5^, Mohd Zulkhairi Mohd Yusoff^1,2,5*^

^1^ Department of Bioprocess Technology, Faculty of Biotechnology and Biomolecular Sciences, Universiti Putra Malaysia, 43400, UPM Serdang, Selangor, Malaysia

[mzulkhairi@upm.edu.my](mailto:mzulkhairi@upm.edu.my), [ar_muhaimin@upm.edu.my](mailto:ar_muhaimin@upm.edu.my), [gs71267@student.upm.edu.my](mailto:gs71267@student.upm.edu.my)

^2^ Laboratory of Biopolymer and Derivatives, Institute of Tropical Forestry and Forest Products (INTROP), Universiti Putra Malaysia, 43400 UPM Serdang, Selangor, Malaysia

[mzulkhairi@upm.edu.my](mailto:mzulkhairi@upm.edu.my), [abdillahfarid@upm.edu.my](mailto:abdillahfarid@upm.edu.my), [gs54370@student.edu.my](mailto:gs54370@student.edu.my)

^3^ Department of Biological Functions Engineering, Graduate School of Life Science and Systems Engineering, Kyushu Institute of Technology, 2-4 Hibikino, Wakamatsu, Kitakyushu, Fukuoka, 808-0196, Japan

[toshi.maeda@life.kyutech.ac.jp](mailto:toshi.maeda@life.kyutech.ac.jp)

^4^ Laboratory of Sustainable Animal Production and Biodiversity, Institute of Tropical Agriculture and Food Security (ITAFoS), Universiti Putra Malaysia, 43400 UPM Serdang, Selangor, Malaysia

[mohdhuzairi@upm.edu.my](mailto:mohdhuzairi@upm.edu.my)

^5^ Department of Agroindustry, Faculty of Agricultural Technology, University of Brawijaya, Veteran St, Malang, East Java 65145, Indonesia

[hendrix@ub.ac.id](mailto:hendrix@ub.ac.id)

***Corresponding author**

Fig. S1. Heatmap of 28 dominant bacterial genera (>0.5% relative abundance) across composting stages (Days 0, 6, 36, and 60).
